# Opportunistic health screening for cardiovascular and diabetes risk factors in primary care dental practices: experiences from a service evaluation and a call to action

**DOI:** 10.1038/s41415-023-6449-6

**Published:** 2023-11-10

**Authors:** Janine Doughty, Simon M. Gallier, Martha Paisi, Robert Witton, Amanda J. Daley

**Affiliations:** 41415370684001 NIHR Clinical Lecturer in General Dental Practice, School of Dentistry, Royal Liverpool University Dental Hospital, Pembroke Place, Liverpool, UK; 41415370684002 Future Health Partnership, Suite 11, 103–105 Harley Street, London, UK; 41415370684003 Research Lead (Peninsula Dental Social Enterprise) and Senior Research Fellow in Public Health, School of Nursing and Midwifery, Faculty of Health, University of Plymouth, Plymouth, UK; 41415370684004 Professor of Community Dentistry, Peninsula Dental School, Faculty of Health, University of Plymouth, Plymouth, UK; 41415370684005 Professor of Behavioural Medicine, Centre for Lifestyle Medicine and Behaviour (CLiMB), The School of Sport, Exercise and Health Sciences, Loughborough University, LE11 3TU, UK

## Abstract

**Introduction** Substantial evidence has established associations between oral health and chronic diseases, mediated by common risk factors and inflammatory processes. Dental professionals are aptly positioned to screen for cardiovascular disease and diabetes high-risk indicators. This article presents a service evaluation of two dental practices delivering health screening in dental practice, and a call to action for the wider profession.

**Methods** Following training, two general dental practices implemented a suite of health screens, including blood pressure, blood glucose, cholesterol, body mass index (BMI) and waist-to-height ratio. A service evaluation was undertaken to review and improve service provision.

**Results** Most patients (78.4%) had blood pressure values above normal range. More than half (55.8%) were outside of the healthy range for BMI. Out-of-range results for cholesterol were observed in less than one-fifth (16.7%). High blood glucose values were observed for few patients (3.3%).

**Conclusion** Dental professionals can be successfully trained to deliver health screening interventions. Out-of-range health screening results offer an opportunity to provide targeted health advice for both the oral and general health. Clear protocols and careful interpretation of screening tests are required to minimise patients' confusion. More robust alliances between dental and general medical care are recommended.

**Supplementary Information:**

Zusatzmaterial online: Zu diesem Beitrag sind unter 10.1038/s41415-023-6449-6 für autorisierte Leser zusätzliche Dateien abrufbar.

## Key points

- This manuscript introduces the concepts of real-world implementation of health screening in NHS and mixed dental practices.
- The paper gives an indication of the training and competencies required to deliver health screening.
- The article illustrates the various opportunities and challenges that present when delivering a health screening service in dental settings.
- The manuscript also highlights what barriers need to be overcome in the future for health screening to be routinely integrated into dental practice.

**Supplementary Information:**

Zusatzmaterial online: Zu diesem Beitrag sind unter 10.1038/s41415-023-6449-6 für autorisierte Leser zusätzliche Dateien abrufbar.

## Introduction

The mouth is an integral part of overall health and wellbeing. The association between oral health and major chronic diseases is now well established and evidenced.^1^^,^^2^^,^^3^ We know with increasing scientific certainty that chronic inflammation in the mouth has negative impacts across the body. Implications for diabetes management and cardiovascular disease (CVD) are two clear examples of this.^2^^,^^4^ Persistent low levels of chronic inflammation in the mouth is the key causative mechanism between oral and general health.^5^ We highlight this evidence because it contributes to the argument that although dental professionals are specialists of the oral cavity and peri-oral regions, with the generation of new knowledge, consideration should be given to expanding scope of practice to include screening for health conditions relevant to oral health.^6^ Health screening also aligns dentistry with the NHS Long Term Plan which encourages collaborative working across NHS platforms for early detection of high-risk conditions associated with CVD.^7^

Studies have consistently shown that dental professionals, including dentists, hygiene therapists and dental nurses, are capable of expanding their scope of clinical practice to include screening for health conditions and are ideally placed to expedite referrals to primary and secondary care services.^8^^,^^9^^,^^10^ Utilising the whole dental health team to deliver health screening could therefore offer the opportunity for dental professionals to enhance their scope of practice and support NHS England's ambitions to maximise the potential of the whole dental health team.^11^ Health screening aligns with the NHS Making Every Contact Count (MECC) aim of upscaling opportunistic delivery of consistent and concise healthy lifestyle information to the public.^12^ MECC promotes the concept that every healthcare professional, including dental professionals, has a role to play in offering the public opportunistic behaviour change advice and support during routine consultations and directing them to relevant local health services.

Studies have highlighted that health screening in dental practices can protect against missed opportunities for screening of at-risk groups (that is, smokers, people with high blood pressure) who infrequently attend general practitioner (GP) services.^13^^,^^14^ This is because every two-year period, half of the population in England access services for dental care.^15^ Therefore, dental appointments in primary and secondary care may offer the opportunity for dental teams to detect chronic disease during the early asymptomatic phases.

The potential implications of health screening in dental practice are reductions in morbidity, mortality, and onward cost to healthcare systems by avoiding acute presentations of late-stage chronic disease.^16^ Therefore, the dental practice may offer an untapped opportunity for screening, offering personalised brief interventions based on screening findings and, where applicable, a gateway for referral and early diagnosis.^17^ However, though patient acceptability of health screening in dental practice has consistently been shown to be high and numerous dental professionals have advocated for its implementation, the translation of research and guidance into dental practice has been stymied by powerful structural challenges.^18^^,^^19^ In England, barriers to health screening in dental settings include issues of scope of practice, challenges in ascertaining whether specific dental indemnification is required to perform screening and absence of commissioning for health screening activities.^18^^,^^20^

Here, we present an exemplar service evaluation of health screening in two general dental practices in England and Wales and thereafter discuss recommendations and a call to action for health screening to take place in dental settings. The aims of the service evaluation were to: 1) evaluate the number of patients who were willing to accept health screening; 2) use screening data to identify areas for improvement in the delivery of health screenings and documentation of these data; 3) iteratively improve health screening service delivery by feeding back findings to the dental teams; and 4) share recommendations from the service evaluation and call to action the wider profession through dissemination activities.

## Methods

### Dental practices and training

Two dental practices in North West England and the Welsh border region contributed data to this service evaluation: one a predominantly NHS practice, and the other a mixed NHS and private practice. A suite of screening tests was offered to dental patients attending for routine dental examination or hygiene appointments. Although the UK National Screening Committee does not recommend population level screening for Type II diabetes or hypertension in adults, with regard to the latter, it identifies the need for and recommends a vascular risk management programme, which subsequently evolved into the NHS Health Check.^21^ The Total Health Screens were in line with NHS Health Check reviews and included assessment of blood pressure, cholesterol, blood glucose, body mass index (BMI) and waist-to-height ratio. NHS Health Checks are designed for healthy adults to detect early signs of CVD and Type II diabetes.^22^ Additionally, these screening tests were selected because of their relationship with oral health and/or common risk factors for oral health complications, for example, diet and chronic inflammation. Smoking status and alcohol consumption were not included in the suite of screens described here as these were already routinely assessed during the history-taking process at both dental practices.

Total Health Screens are an organisation which provide health screening training and resources to for dental practices.^23^ The practice principals at both sites had employed the services of Total Health Screens and were in support of the intervention. All members of the dental team at each practice attended health screening training sessions. The training sessions provided information about the physiology of chronic health conditions; the relevance of health screening for general health and links with oral health; clinical boundaries of screening results (in and out of range); hands-on instructions on how to perform testing; scenario-based role plays; protocol for managing patients with out-of-range results (including scripts and frequently asked questions); and strategies for improving health. The introductory training session lasted one hour. A second training session encompassed didactic teaching and hands-on training which lasted two hours. At practice two, a further two-hour refresher session was undertaken.

### Patients

The opportunity to participate in health screening was advertised to patients using posters in the waiting area and/or was verbally offered during an appointment with an appropriately trained dental professional. Patients were informed about the purposes of testing (that is, to detect any out-of-range results that could be indicate a risk of CVD and diabetes) and advised about the links between oral health and chronic disease. Written consent for the procedures was not required as screening was performed as an integrated part of dental service provision.

### Service description

Each practice implemented health screening using approaches that best suited their model of dental service delivery (details are provided in the online Supplementary Information).^24^ Dental patients at both practices were offered the suite of health screens described above. Blood pressure screening was performed using an electronic sphygmomanometer (that is, blood pressure cuff). Screening for blood sugar, cholesterol and triglycerides were performed using handheld Accutrend Plus point-of-care finger prick devices. The devices have demonstrated high sensitivity (glucose = 80.0%; triglycerides = 90.5%; total cholesterol = 84.4%) and specificity (glucose = 100.0%; triglycerides = 96.9%; total cholesterol = 95.2%).^25^ Though the units tend toward overestimating glucose and triglyceride levels when compared with laboratory results, they are being used for screening, not definitive diagnostic purposes, and thus are considered a valid alternative tool for monitoring metabolic syndrome and cardiovascular risk factors. BMI and waist-to-height ratio were performed using scales and tape measure. The screening tests were offered, performed, and the results fed back to patients within the same appointment. Practice two performed high density lipoprotein (HDL) and non-HDL cholesterol tests, as opposed to overall cholesterol value, and did not offer height-to-waist ratio.

Screening was offered during a routine dental examination appointment at practice one and annually during a hygiene appointment at practice two. Standard medical reference ranges for the screening tests can be found in Table 1. At practice one, health screening was offered by dental nurses in a separate health screening room before a regular examination appointment and at practice two, hygiene therapists offered testing in the dental surgery following routine hygiene appointments. All patients whose results were out of range were verbally advised to attend their GP for follow-up.

**Table 1:** Reference ranges for screening tests

Screening test	Outcome	Values
Blood pressure	Hypotension	Less than 80/less than 60
	Healthy range	80-120/60-80
	Prehypertension	121-139/81-89
	High blood pressure	140-159 or higher/90-99 or higher
	High blood pressure crisis	Higher than 180/higher than 120
Total cholesterol	Healthy range	5 or below
	HDL healthy range	1 or above
	Non-HDL healthy range	4 or below
Blood glucose	Low blood glucose	Below 4
	Healthy range	4.0-5.4 (fasting) up to 7.8 (post prandial)
	High blood sugar	Above 7.8
BMI	Underweight	Below 18.5
	Healthy range	18.5-24.9
	Overweight	25.0-29.9
	Obese	30.0 and above
Waist-height ratio	Healthy range	0.42-0.49 (women) 0.42-0.53 (men)

### Data collection and analysis

The health screening data was collected from patients at practice one between 31 August 2020 and 8 November 2021, and at practice two between 1 February 2021 and 31 January 2023. Routine screening data were collected from patients, for example, age (years), sex and the outcomes of the health screening tests. In both practices, screening outcomes were documented in the body of the clinical notes.

Anonymised aggregated data were collated to inform the findings of this service evaluation; therefore, the data were sufficiently anonymised to prevent re-identification of patients or to incur any breaches of patient confidentiality and are thus exempt from the requirement of individual informed consent processes.^26^Data were analysed using descriptive statistics. The Medical Research Council and NHS Research Authority decisions tool was used to determine that the service evaluation would not be considered research by the NHS and therefore did not require ethical approval.

## Results

There were 11,200 patients who attended practice one during the screening period, of whom 458 (4.1%) were offered and accepted screening. At practice two, there were 871 patients signed up to the membership scheme, which includes health screening: of these, 57 (6.5%) had been offered and accepted health screening. Errors in data recording were uncommon but occurred most frequently for those screening tests requiring a calculation, that is, BMI (0.8% errors) and waist-to-height ratio (1.7% errors). Missing data for each variable ranged from 1.5-26.2%. Over one-quarter (26.2%) of patients at practice one did not have a value for waist-to-height ratio. The findings from the screening practices indicated that both male and female patients accepted health screening, the majority of whom (86.4%) were aged 30 years and over, with a mean age of 52.5 ± 15.8 (Table 2).

**Table 2:** Characteristics of patients who accepted health screening

Characteristics	Practice one	Practice two	Total
	n (%) n = 458	n (%) n = 57	n (%) n = 525
**Sex**			
Male	232 (50.7)	26 (45.6)	258 (50.1)
Female	226 (49.3)	31 (54.4)	257 (49.9)
Missing/unknown	0 (0)	0 (0)	0 (0)
**Age (years)**			
18-29	53 (11.6)	4 (7.0)	57 (11.1)
30-39	66 (14.4)	10 (17.5)	76 (14.8)
40-49	66 (14.4)	5 (8.8)	71 (13.8)
50-59	87 (19.0)	11 (19.3)	98 (19.0)
60-69	89 (19.4)	13 (22.8)	102 (19.8)
70+	84 (18.3)	14 (24.6)	98 (19.0)
Missing/unknown	11 (2.4)	0 (0)	11 (2.1)
Data entry error	2 (0.4)	0 (0)	2 (0.4)
Mean age (+/- SD) years	52.1 +/- 16.9	55.7 +/- 16.5	52.5 +/- 15.8

At both practices, prehypertension and high blood pressure were common (Table 3). Of all patients screened, most (78.4%) had blood pressure values above normal range and one-third (33.2%) had blood pressure values indicating hypertension, that is, 140-159 or higher systolic/90-99 or higher diastolic. The mean blood pressure values were 132.1 (± 18.8) systolic/85.7 (± 13.6) diastolic.

**Table 3:** Health screening outcomes

Health screening outcome	Practice one	Practice two	Total
	n (%) n = 458	n (%) n = 57	n (%) n = 525
**Blood pressure**			
Normal range	94 (20.5)	10 (17.5)	104 (20.2)
Prehypertension	213 (46.5)	20 (35.1)	233 (45.2)
High blood pressure	144 (31.4)	27 (47.4)	171 (33.2)
Missing/unknown	7 (1.5)	0 (0)	7 (1.4)
Data entry error	0 (0)	0 (0)	0 (0)
**Mean blood pressure (+/- SD)**			
Systolic	132.3 +/- 18.5	133 +/- 13.6	132.1 +/- 18.8
Diastolic	86.1 +/- 13.5	84.2 +/- 11.2	85.7 +/- 13.6
**Cholesterol**			
Total cholesterol normal range	362 (79.0)	50 (88.8)	412 (80.0)
Above normal range	79 (17.2)	7 (12.3)	86 (16.7)
Missing/unknown	15 (3.3)	0 (0)	15 (2.9)
Data entry error	2 (0.4)	0 (0)	2 (0.4)
Mean total cholesterol (+/- SD)	5.4 +/- 2.3	4.5 +/- 1.2	5.3 +/- 5.3
Mean HDL (+/- SD)	-	1.6 +/- 0.4	-
Mean non-HDL (+/- SD)	-	2.5 +/- 1.1	-
**Blood glucose**			
Below normal range	231 (50.4)	0 (0.0)	231 (44.9)
Normal range	208 (45.4)	49 (86.0)	257 (49.9)
Above normal range	9 (2.0)	8 (14.0)	17 (3.3)
Missing/unknown	9 (2.0)	0 (0)	9 (1.7)
Data entry error	1 (0.2)	0 (0)	1 (0.2)
Mean blood glucose (+/- SD)	4.0 +/- 1.4	6.7 +/- 1.8	4.4 +/- 2.3
**BMI**			
Underweight	5 (1.1)	0 (0)	5 (1.0)
Healthy range	198 (43.2)	12 (21.1)	210 (40.8)
Overweight	168 (36.7)	27 (47.4)	195 (37.9)
Obese	74 (16.2)	18 (31.6)	92 (17.9)
Missing/unknown	9 (2.0)	0 (0)	9 (1.7)
Data entry error	4 (0.8)	0 (0)	4 (0.8)
Mean BMI (+/- SD)	25.8 +/- 4.9	27.6 +/- 4.1	25.9 +/- 5.2
**Waist-height ratio**			
Under normal range	11 (2.4)	-	11 (2.4)
Normal range	137 (29.9)	-	137 (29.9)
Above normal range	182 (39.7)	-	182 (39.7)
Missing/unknown	120 (26.2)	-	120 (26.2)
Data entry error	8 (1.7)	-	8 (1.7)
Mean waist-height (+/- SD)	0.58 +/- 1.02	-	0.58 +/- 1.02

Out-of-range results for cholesterol were observed in less than one-fifth (16.7%) of patients. Mean cholesterol values were 5.3 (± 5.3). Practice two also screened for HDL and non-HDL cholesterol, where mean values were within healthy range: 1.6 (± 0.4) and 2.5 (± 1.1), respectively. Low blood glucose values were obtained for almost half (44.9%) of patients undergoing screening. High blood glucose values were observed for a small number of patients (3.3%). Mean blood glucose was within the healthy range (4.4 ± 2.3). More than half (55.8%) of patients were outside of the healthy range for BMI and most were living with overweight (37.9%) or obesity (17.9%). Mean BMI was within the overweight range (25.9 ± 5.2). Of the 330 patients who had accurate entries for height-to-waist measurement from practice one, more than half (55.1%) had above normal range values and the mean value was above normal range (0.58 ± 1.02).

## Discussion

We have outlined a rationale for integrating health screening within routine primary care dental consultations. We have presented a case exemplar describing two practices that provided a suite of health screening tests of relevance to oral health. Health screening was commonly accepted by patients aged 50 years or over. A significant proportion of patients had out-of-normal-range values for their blood pressure, BMI and waist-height ratio. Most patients had blood pressure values outside of the healthy range. Less than half had BMI within a healthy range and more than half of patients were living with overweight or obesity. Most patients had blood glucose values which were within or below healthy range and many had within-range cholesterol values. Mean values for cholesterol, blood pressure, BMI and height-to-waist ratio were above-normal-range values. Although mean values for blood glucose were within healthy range values, almost half of patients had a blood glucose value below normal range.

The proportion of patients with hyperglycaemia in this service evaluation (2.0-14.0%) was consistent with findings of a recent systematic review of diabetes screening in dental settings (1.7-24.0%). In the UK, as many as 11% of the adult population have been reported to have impaired glucose regulation.^27^ Additionally, almost half of dental patients aged ≥45 years are at risk of developing diabetes in the next ten years.^28^ These figures indicate the positive contribution and impact that screening for diabetes in dental settings may have for early Type II diabetes intervention.^29^ In 2022, new National Institute for Health and Care Excellence guidance advised that, when adults with diabetes attend their annual diabetic review with their GP, they are informed of the higher risk of periodontal disease, the importance of risk-based regular oral health review, and the relationship between periodontitis and blood sugar control. These guidelines are likely to have subsequent impact on referrals of diabetic people into dental practice.

Despite the proposed benefits, universal health screening at the dental practice is not without risk. If screening at the dentist is not performed accurately, there is a risk of burdening the already stretched health services with false-positive results.^30^ Furthermore, there is a possibility that patients with out-of-range screening results do not follow-up with their GP (especially when a dental-GP referral network has not been established), undermining the usefulness of screening at the dental setting.

Out-of-range findings must be interpreted with full understanding of their significance in relation to the context of the dental setting. For example, social factors that may affect blood glucose readings could lead patients to delay meals until after they have attended the dentist to avoid having debris in their teeth; this could account for the high proportion of patients with lower-than-normal-range blood sugar. A further discussion between dental professional and patient is warranted to explain the clinical significance of low blood sugar readings and one solution may be to encourage patients to eat one hour before coming to their dental appointment.

In this service evaluation, almost half of patients who underwent screening had blood pressure values in the range of prehypertension, similar to screening interventions undertaken in other hypertension screening studies in dental settings.^31^ Among the UK population, 11% of women and 14% of men are living with uncontrolled hypertension. Literature suggests that white coat hypertension can affect as much as one-third of patients, with an elevated blood pressure in a clinical environment.^32^ Further, dental anxiety can precipitate transient hypertension.^33^^,^^34^ Even patients who appear calm in the dental setting have been reported to have elevated blood pressure measurements indicative of significant physiological distress.^35^ The implications of this are that unnecessary referrals may be made to GPs. A way to mitigate this issue could be to perform health screening at the end of appointments once dental treatment is complete and allow a few minutes for the patient to physiologically recover before health screening takes place.

A small but significant number of patients had a blood pressure measure within the range of a hypertensive emergency. Individual-level data from clinical notes were not used in this service evaluation; therefore, it is uncertain whether patients with severe hypertension were followed-up by the practice to ensure that they had accessed emergency care. Future iterations of health screening in dental settings would benefit from clear protocol to follow in the event of medical emergencies to ensure the safety and wellbeing of patients.

Hypercholesterolaemia was detected in 16.7% of the patient population in this evaluation, lower than the prevalence in the UK adult population (23.5%).^36^ Detection of hypercholesterolaemia may be an important motivator for patients as the link between atherosclerotic CVD becomes increasingly well-evidenced.^37^

The findings for BMI values reflect similar proportions to that of the general UK population, where as many as 42.5% of the adult population are in the overweight and 24.5% in the obese ranges.^38^ However, at practice one, 43.2% of patients were in the healthy range, compared to 32.5% in the general UK population. This finding may indicate that the subset of the UK population who regularly attend dental appointments and who accept health screening are different in some way to the general population, for example, greater health agency or focus on preventative health.

## Moving towards routine health screening in dental settings

An independent health economic review, commissioned by The Office of the Chief Dental Officer (England), estimated that £48,204,486 could be saved over a three-year period if primary care NHS dental providers in England were to fully engage in case finding for people aged 40 years and older with previously undetected risk factors for CVD.^16^ However, for health screening activity to translate into policy and subsequently into practice, there must be fertile ground for its implementation. Necessary assurances include confirmation of indemnity requirements for dental professionals who choose to deliver such interventions and a clear position of the professional regulators on health screening and dental professionals' scope of practice. Outside of dentistry, other healthcare organisations and statutes provide a clear stance on the regulation of health screening. For example, the Care Quality Commission excludes from regulation 'blood tests carried out by means of a pinprick test or removing blood from a vein where the sample is not sent to a laboratory for analysis'.^39^ Under the Medical Act 1983, health screening that does not lead to formal diagnosis or treatment of a condition is not considered a reserved procedure.^40^ At the time of writing the published dental contracting frameworks do not include commissioning provisions for health screening activity undertaken in dental settings.^41^ Nevertheless, this service evaluation has demonstrated that dental practitioners could play an important role in identifying patients who may be in need of clinical intervention to prevent or delay CVD and diabetes progression.

Studies exploring patient experiences of NHS health checks in healthcare settings have reported that most patients believe that they have benefited by mechanisms such as reassurance and wake-up calls reinforcing the importance of healthy lifestyles for optimal health.^42^ However, some people report confusion and frustration about the implications of their screening results and follow-up actions.^43^ To protect patients and ensure maximal benefit from health screening in dental settings, clear and concise protocols for interpretation, follow up and tailored health advice are needed. Training of dental professions to have appropriate conversations with patients regarding their results is also necessary. For example, skills in raising the topic of weight and BMI appropriately would be particularly important. Additionally, strong interprofessional collaboration between dental and general medical practice at a local/regional level is needed to ensure judicious use of resources and appropriate signposting of patients for support and clinical intervention if and when required. The ambition of an alliance between dental professionals and GPs would ultimately be to reduce healthcare costs through early detection of disease and access to treatment. The European Federation of Periodontology has recently stressed the importance of collaboration between dental professionals and family doctors to treat their patients' overall health.^44^ However, in England, there are workforce shortages in general medical practice. Health screening in dental settings should aim to alleviate rather than add further burden to overstretched healthcare professionals, including GPs, nurses and healthcare professionals.^45^ Offering tailored health advice and agreeing appropriate referral criteria with local GP input is essential to ensuring that patients access timely and appropriate care when it is needed and feel empowered to make healthful behaviours changes.

The treadmill effect of units of dental activity targets under the current NHS dental contract poses a significant barrier to health screening within NHS-funded dental practices. In 2021, the contract came under fire again from the profession, and petitions have revealed appetite for systemic change.^46^ Further, due to the limitations of the current contract, many dental professionals are making plans to move from the NHS to providing private dental care.^47^ Health screening therefore may struggle to find a home within NHS dental practices unless structural barriers are addressed. To fully realise the advantages that health screening in dental settings offers for the NHS Core20PLUS5 population groups and avoid widening of health inequalities, finding ways to support its integration into NHS dental practices is crucial.^48^ To move toward widespread adoption of health screening in dental practice, the responsibilities of the dental team for the broader health needs of dental patients must be recognised and be underpinned by a dental contract that enables the realisation of oral health as an integral part of the general health and wellbeing agenda.

## Conclusion

Health screening in dental settings offers novel opportunities to detect a substantial number of patients with risk factors for chronic disease which have implications for oral health and vice versa. Dental professionals can be successfully trained to deliver the screening interventions and are aptly placed for delivering brief lifestyle advice and signposting patients to general medical care or other appropriate clinical services. However, clear protocols and careful interpretation of screening tests are required to minimise patients' confusion and frustration and more robust alliances between dental and general medical care are recommended.

### Supplementary Information

Supplementary Information (PDF 125KB)

## References

[1] Daly B, Thompsell A, Sharpling J *et al.* Evidence summary: the relationship between oral health and dementia. *Br Dent J* 2018; **223:** 846-853. 10.1038/sj.bdj.2017.992 29192686

[2] Dietrich T, Webb I, Stenhouse L *et al.* Evidence summary: the relationship between oral and cardiovascular disease. *Br Dent J* 2017; **222:** 381-385. 10.1038/sj.bdj.2017.224 28281612

[3] Manger D, Walshaw M, Fitzgerald R *et al.* Evidence summary: the relationship between oral health and pulmonary disease. *Br Dent J* 2017; **222:** 527-533. 10.1038/sj.bdj.2017.315 28387268

[4] D'Aiuto F, Gable D, Syed Z *et al.* Evidence summary: the relationship between oral diseases and diabetes. *Br Dent J* 2017; **222:** 944-948. 10.1038/sj.bdj.2017.544 28642531

[5] Eisenstein M. Homing in on an oral link to inflammatory disease. *Nature* 2021; DOI: 10.1038/d41586-021-02918-4. 10.1038/d41586-021-02918-4 34707270

[6] Daley A J. Time to get our teeth into reducing obesity: should dentists screen and deliver interventions to reduce obesity in the population? *Br Dent J* 2022; **232:** 78-79. 10.1038/s41415-022-3872-z PMC8796197 35091605

[7] NHS. The NHS Long Term Plan.2019.Available at https://www.longtermplan.nhs.uk/wp-content/uploads/2019/08/nhs-long-term-plan-version-1.2.pdf (accessed October 2023).

[8] Genco R J, Schifferle R E, Dunford R G, Falkner K L, Hsu W C, Balukjian J*.* Screening for diabetes mellitus in dental practices: A field trial. *J Am Dent Assoc* 2014; **145:** 57-64. 10.14219/jada.2013.7 24379330

[9] Wright D, Muirhead V, Weston-Price S, Fortune F. Type 2 diabetes risk screening in dental practice settings: a pilot study. *Br Dent J *2014; **216:** E15. 10.1038/sj.bdj.2014.250 24722119

[10] Suarez-Durall P, Osborne M S, Enciso R, Melrose M D, Mulligan R. Results of offering oral rapid HIV screening within a dental school clinic. *Spec Care Dentist* 2019; **39:** 188-200. 10.1111/scd.12363 30719739

[11] NHS England. Building dental teams: Supporting the use of skill mix in NHS general dental practice - long guidance. 2023. Available at https://www.england.nhs.uk/long-read/building-dental-teams-supporting-the-use-of-skill-mix-in-nhs-general-dental-practice-long-guidance/ (accessed October 2023).

[12] Public Health England. Making Every Contact Count (MECC): Consensus statement. 2016. Available at https://www.england.nhs.uk/wp-content/uploads/2016/04/making-every-contact-count.pdf (accessed October 2023).

[13] Chung R, Leung S-Y J, Abel S N *et al.* HIV screening in the dental setting in New York State. *PLoS One* 2020; DOI: 10.1371/journal.pone.0231638. 10.1371/journal.pone.0231638 PMC7161960 32298336

[14] Ireland R S, Bowyer V, Ireland A, Sutcliffe P. The medical and dental attendance pattern of patients attending general dental practices in Warwickshire and their general health risk assessment. *Br Dent J* 2012; **212:** E12. 10.1038/sj.bdj.2012.313 22538925

[15] NHS Digital. NHS Dental Statistics for England - 2019-20 Annual Report. 2020. Available at https://digital.nhs.uk/data-and-information/publications/statistical/nhs-dental-statistics/2019-20-annual-report (accessed October 2023).

[16] Shivji S. Alone we are strong, together we are stronger. 2022. Available at https://blogs.bmj.com/bmjleader/2022/10/05/alone-we-are-strong-together-we-are-stronger-by-shabir-shivji/?utm_campaign=shareaholic&utm_medium=twitter&utm_source=socialnetwork (accessed October 2023).

[17] Santella A J, Conway D I, Watt R G. The potential role of dentists in HIV screening. *Br Dent J* 2016; **220:** 229-233. 10.1038/sj.bdj.2016.172 26964593

[18] Friman G, Hultin M, Nilsson G H, Wårdh I. Medical screening in dental settings: a qualitative study of the views of authorities and organisations. *BMC Res Notes* 2015; **8:** 580. 10.1186/s13104-015-1543-8 PMC4610051 26478099

[19] Creanor S, Millward B A, Demaine A *et al.* Patients' attitudes towards screening for diabetes and other medical conditions in the dental setting. *Br Dent J* 2014; **216:** E2. 10.1038/sj.bdj.2013.1247 24413142

[20] Feinstein-Winitzer R T, Pollack H A, Parish C L, Pereyra M R, Abel S N, Metsch L R*.* Insurer views on reimbursement of preventive services in the dental setting: results from a qualitative study. *Am J Public Health* 2014; **104:** 881-887. 10.2105/AJPH.2013.301825 PMC3987575 24625150

[21] UK Government. UK NSC Recommendations. Available at https://view-health-screening-recommendations.service.gov.uk/ (accessed October 2023).

[22] NHS. NHS Health Check. Available at https://www.nhs.uk/conditions/nhs-health-check/ (accessed October 2023).

[23] Total Health Screens. Available at https://www.totalhealthscreens.com/ (accessed October 2023).

[24] Hoffmann T C, Glasziou P P, Boutron I *et al.* Better reporting of interventions: template for intervention description and replication (TIDieR) checklist and guide. *BMJ* 2014; **348:** g1687. 10.1136/bmj.g1687 24609605

[25] Coqueiro R S, Santos M C, Neto J S, Queiroz B M, Brügger N A, Barbosa A R*.* Validity of a portable glucose, total cholesterol, and triglycerides multi-analyser in adults. *Biol Res Nurs* 2014; **16:** 288-294. 10.1177/1099800413495953 23871994

[26] NHS East London NHS Foundation Trust. Consent to use data. Available at https://www.elft.nhs.uk/research/conducting-study/consent-use-data (accessed October 2023).

[27] Moody A, Cowley G, Fat L N, Mindell J S. Social inequalities in prevalence of diagnosed and undiagnosed diabetes and impaired glucose regulation in participants in the Health Surveys for England series. *BMJ Open* 2016; DOI: 10.1136/bmjopen-2015-010155. 10.1136/bmjopen-2015-010155 PMC4746474 26857106

[28] Bould K, Scott S E, Dunne S, Asimakopoulou K. Uptake of screening for type 2 diabetes risk in general dental practice; an exploratory study. *Br Dent J* 2017; **222:** 293-296. 10.1038/sj.bdj.2017.174 28232690

[29] Yonel Z, Cerullo E, Kröger A T, Grey L J. Use of dental practices for the identification of adults with undiagnosed type 2 diabetes mellitus or non-diabetic hyperglycaemia: a systematic review. *Diabet Med* 2020; **37:** 1443-1453. 10.1111/dme.14324 32426909

[30] Doble A, Bescos R, Witton R, Shivji S, Ayres R, Brookes Z*.* A Case-Finding Protocol for High Cardiovascular Risk in a Primary Care Dental School-Model with Integrated Care. *Int J Environ Res Public Health* 2023; **20:** 4959. 10.3390/ijerph20064959 PMC10049228 36981868

[31] Abdulwahab M, Kamal M, Akbar A. Screening for High Blood Pressure at the Dentist's Office. *Clin Cosmet Investig Dent* 2022; **14:** 79-85. 10.2147/CCIDE.S358890 PMC8986194 35399622

[32] Nuredini G, Saunders A, Rajkumar C, Okorie M. Current status of white coat hypertension: where are we? *Ther Adv Cardiovasc Dis* 2020; DOI: 10.1177/1753944720931637. 10.1177/1753944720931637 PMC7318827 32580646

[33] Humphris G M, Freeman R, Campbell J, Tuutti H, D'Souza V. Further evidence for the reliability and validity of the Modified Dental Anxiety Scale. *Int Dent J* 2000; **50:** 367-370. 10.1111/j.1875-595x.2000.tb00570.x 11197195

[34] Humphris G, King K. The prevalence of dental anxiety across previous distressing experiences. *J Anxiety Disord *2011; **25:** 232-236. 10.1016/j.janxdis.2010.09.007 20952156

[35] Liau F L, Kok S-H, Lee J-J *et al.* Cardiovascular influence of dental anxiety during local anaesthesia for tooth extraction. *Oral Surg Oral Med Oral Pathol Oral Radiol Endod* 2008; **105:** 16-26. 10.1016/j.tripleo.2007.03.015 17656135

[36] Bilitou A, Were J, Farrer A *et al.* Prevalence and Patient Outcomes of Adult Primary Hypercholesterolemia and Dyslipidemia in the UK: Longitudinal Retrospective Study Using a Primary Care Dataset from 2009 to 2019. *Clinicoecon Outcomes Res* 2022; **14:** 189-203. 10.2147/CEOR.S347085 PMC8994561 35411162

[37] Gianos E, Jackson E A, Tejpal A *et al.* Oral health and atherosclerotic cardiovascular disease: A review. *Am J Prev Cardiol* 2021; **7:** 100179. 10.1016/j.ajpc.2021.100179 PMC8387275 34611631

[38] Kivimäki M, Strandberg T, Pentti J *et al.* Body-mass index and risk of obesity-related complex multimorbidity: an observational multicohort study. *Lancet Diabetes Endocrinol* 2022; **10:** 253-263. 10.1016/S2213-8587(22)00033-X PMC8938400 35248171

[39] Care Quality Commission. Scope of registration: Regulated activities. Diagnostic and screening procedures. 2022. Available at https://www.cqc.org.uk/guidance-providers/scope-registration-regulated-activities#diagnostic-screening-services (accessed October 2023).

[40] UK Government. *The Medical Act 1983.* London: The Stationary Office, 1983.

[41] NHS England. Standard General Dental Services Contract - July 2018. 2018. Available at https://www.england.nhs.uk/wp-content/uploads/2018/08/general-dental-services-contract-2018.pdf (accessed October 2023).

[42] Usher-Smith J A, Harte E, MacLure C *et al.* Patient experience of NHS health checks: a systematic review and qualitative synthesis. *BMJ Open* 2017; DOI: 10.1136/bmjopen-2017-017169. 10.1136/bmjopen-2017-017169 PMC5724113 28801437

[43] Riley B L, MacDonald J, Mansi O *et al.* Is reporting on interventions a weak link in understanding how and why they work? A preliminary exploration using community heart health exemplars. *Implementat Sci* 2008; **3:** 27. 10.1186/1748-5908-3-27 PMC2413262 18492247

[44] Herrera D, Sanz M, Shapira L *et al.* Association between periodontal diseases and cardiovascular diseases, diabetes and respiratory diseases: Consensus report of the Joint Workshop by the European Federation of Periodontology (EFP) and the European arm of the World Organisation of Family Doctors (WONCA Europe). *J Clin Periodontol* 2023; **50:** 819-841. 10.1111/jcpe.13807 36935200

[45] Ungoed-Thomas J. GPs in England treat up to three times more patients than safety limit demands. *The Observer* (London) 2022 November 20.

[46] UK Government and Parliament. Independent review of the NHS dental contract*.* 2021. Available at https://petition.parliament.uk/petitions/564154 (accessed October 2023).

[47] British Dental Association. Nearly half of dentists severing ties with NHS as government fails to move forward on reform. 2022. Available at https://bda.org/news-centre/press-releases/Pages/nearly-half-of-dentists-severing-ties-with-nhs.aspx (accessed October 2023).

[48] NHS England. Core20PLUS5 (adults) - an approach to reducing healthcare inequalities. Available at https://www.england.nhs.uk/about/equality/equality-hub/national-healthcare-inequalities-improvement-programme/core20plus5/ (accessed October 2023).

