# Supplementary material for: Opportunistic health screening for cardiovascular and diabetes risk factors in primary care dental practices: experiences from a service evaluation and a call to action
Source: Br Dent J. 2023 Nov 10;235(9):727–33. doi: 10.1038/s41415-023-6449-6 (PMC10635822; doi:10.1038/s41415-023-6449-6)
Supplement: Supplementary file 1 — Supplementary Information (PDF 125KB) [file 41415_2023_6449_MOESM1_ESM.pdf]

Supplemental material

Intervention mapping using TIDiER checklist

**Intervention components**	Practice 1 Wales	Practice 2 Northwest England
What (materials, information, training)	Two-part training programme taking place over two lunch time sessions.	Two-part training programme taking place over two lunch time sessions. Additional full afternoon hands-on and didactic teaching session with role play.
What (procedures, activities or processes)	Point of care measurements of blood glucose, cholesterol, BMI and height waist ratio. Patients with results outside of normal range advised to attend GP.	Point of care measurements of blood glucose, HbA1c, total cholesterol, LDL and HDL cholesterol, BMI and height waist ratio. Patients with results outside of normal range advised to attend GP.
Who provided	Dental nurses	Hygiene therapists
Who received	All patients	All practice membership patients
How (modes of delivery)	Before routine examination appointment.	Delivered during routine hygiene appointment.
Where	Health screening room	Patients informed of health screening at reception, health screening takes place in surgery
When and how much	Annual health screening at examination appointments.	Annual health screening at regular hygiene visit. Screening performed at the end of the hygiene session.
Tailoring (personalisation or adaptation)	Specific screening tests are used or avoided based on patient wishes.	Specific screening tests are used or avoided based on patient wishes.
Modifications to intervention delivery	None	Initial health screening was provided as an add on cost to hygiene appointments. Later, the health screening was incorporated into the practice membership plan monthly fee and offered to all members.
How well (to what degree has the	Some data entry errors and incorrect calculations for	No data entry errors or incorrect calculations. Intervention intended to for all membership patients as part of

intervention been delivered planned?)	measurement of BMI and height waist ratio.	their plan; however, only accepted by 6.5% of patients. Uncertainty as to whether this occurred because of low intervention fidelity or low acceptability.
